# Correction: Oxidation of Helix-3 Methionines Precedes the Formation of PK Resistant PrPSc

**DOI:** 10.1371/journal.ppat.1006293

**Published:** 2017-05-03

**Authors:** Tamar Canello, Kati Frid, Ronen Gabizon, Silvia Lisa, Assaf Friedler, Jackob Moskovitz, María Gasset, Ruth Gabizon

The authors would like to correct Fig 4, as changes were made to this figure in preparation for publication that were not indicated in the original figures or figure legends. Specifically, the original mAB 6H4/Sc(RML) Mo panel underwent contrast changes that were not indicated in the original figure or figure legend. The corrected Fig 4 includes a rerun experiment without contrast changes. The authors confirm that these changes do not alter their findings.

**Fig 4:**
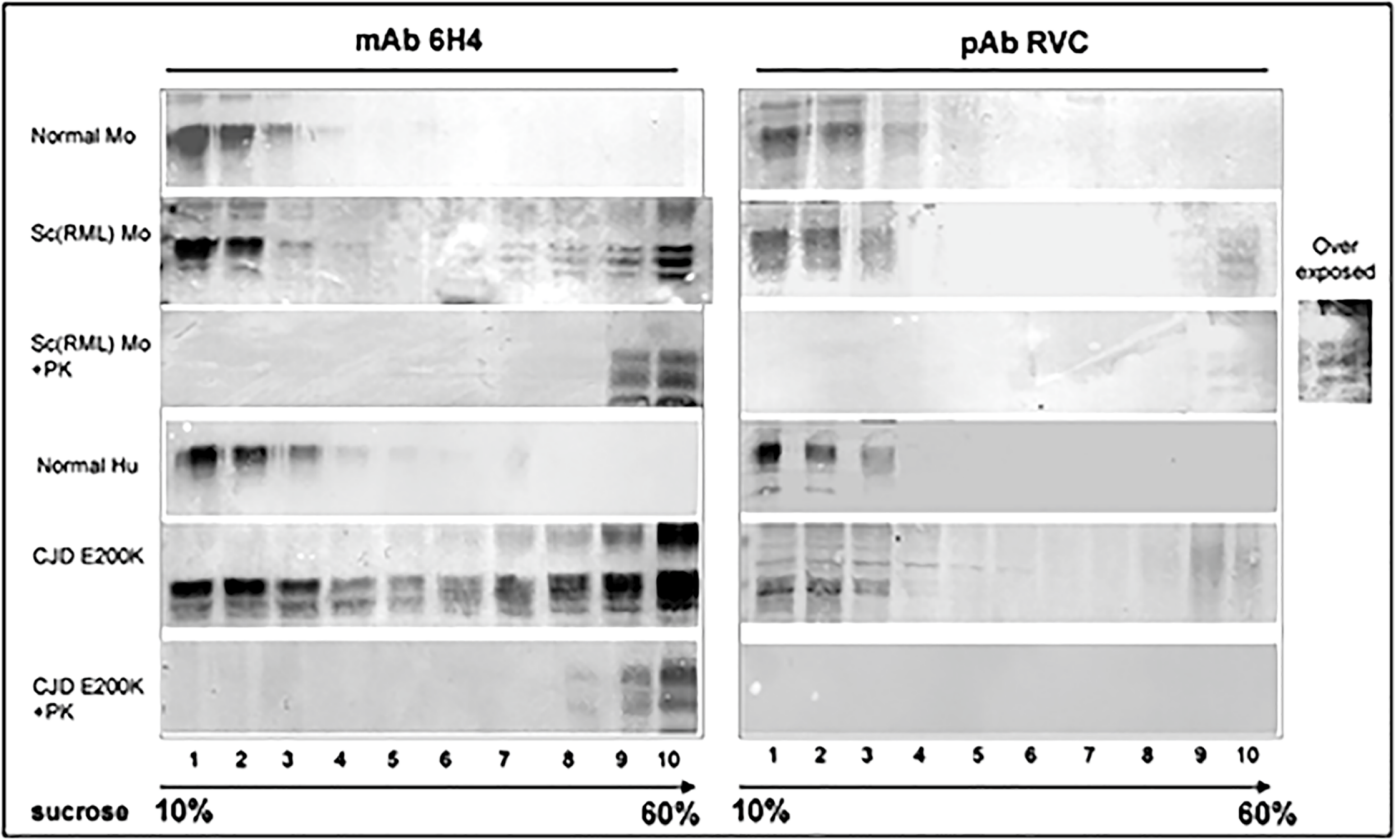
Intermediate PrP forms are oxidized as PrP^Sc^. Sarkosyl extracted brain samples from normal and prion infected mice and humans were subjected to sucrose gradient centrifugation. Fractions from these gradients were digested in the presence or absence of proteinase K and immunoblotted with both mAb 6H4 and pAb RVC. As shown in the previous figures of this manuscript, pAbRVC does not recognize oxidized PrP.

The authors have provided raw, uncropped blots for all panels included in Fig 4 as Supporting Information. The raw, uncropped blots for the following panels are from rerun experiments: mAB 6H4/Normal Mo, mAB 6H4/Sc(RML) Mo + Pk. The raw, uncropped blots for the following panels are from the original experiments: mAB 6H4/Normal Hu, mAB 6H4/CJD E200K, mAB 6H4/CJD E200K + PK, pAB RVC/Normal Mo, pAB RVC/Sc(RML) Mo, pAB RVC/Sc(RML) Mo + PK, pAB RVC/Normal Hu, pAB RVC/ CJD E200K, pAB RVC/CJD E200K + PK.

In addition, Figures 2A, 2B, 2D, 3A, 3B, 5B and 5C underwent a cut and transfer before incubation that was not previously indicated in the figure legends. The corrected figure legends are included here for reference, and original raw gels post cut and transfer are also included as Supporting Information. Original raw blots are also included for Figures 2C and 5A.

**Figure 2**. Testing for the activity of anti-Helix-3 antibodies: pAb RVC does not recognize PrPSc generated in prion infected brains. **(A)** Activity of pAb RVC and RTC, as compared to the established mAb 6H4, against normal brain homogenates from bovine, mouse, humans and hamster. **(B)** Human and mouse normal brain homogenates were immunoblotted with pAb RVC and RTC alone or in the presence of diverse Helix-3 PrP peptides (see Figure 1C for the peptide sequences). **(C)** Brain homogenates from mice, hamster and humans (normal, prion-infected and prioninfected digested with proteinase K), were immunoblotted with mAb 6H4, with pAb RTC or RVC, or with rec Ab R1. **(D)** Mouse scrapie (RML) and human CJD brains (E200K) were digested with PK, processed for MMA reduction as described in the methods and immunoblotted with a PrP mAb 6H4, IPC2, or pAb RVC. For panels A, B and D, the same brain samples (Hu Mo or Bo) were run in consecutive sequences in big gels which were cut after transfer for incubation with different antibodies and peptides incubation, as described in the manuscript.

**Figure 3**. pAb RVC does not recognize PrPSc generated in prion infected cells. **(A)** Brain (normal, scrapie infected, as well as scrapie infected digested with PK) as well as normal and prion-infected cells, (N2a and GT1 infected either with the RML or the 22L prion strains) were extracted and immunoblotted with mAb IPC1 as compared to pAb RVC. **(B)** Effect of the MMA chemical reduction of proteinase K digested ScGT1 and Sc N2a cells on the PrP recognition by mAb IPC1, mAb IPC2 and pAb RVC. For all mouse part in panel A and for all samples in panel B, samples were run in consecutive sequences in big gels which were cut after transfer for incubation with different antibodies and peptides incubation, as described in the manuscript.

**Figure 5**. HuPrP E200K is spontaneously oxidized. **(A)** Brain samples from scrapie infected mice and from humans suffering from familial E200K or sporadic CJD, were digested in the presence or absence of proteinase K and subsequently immunoblotted with mAb 6H4 or pAb RGM. The last 2 lanes of each gel comprise normal GT1 and proteinase K digested ScGT1 cells expressing a chimera Mo/Ha PrP form. **(B)** Human and mouse normal brain homogenates were immunoblotted with the RGM antibody alone or preincubated with several PrP peptides in the Helix-3 Met area. **(C)** Immunoblots of HuPrP(23–230) wt and E200K with 3F4 (recognizing the 109–112 region), DZS18 (recognizing oxidized Met residues in different proteins), IPC2 (recognizing non-oxidized M213) and RGM (recognizing non-oxidized M206). Blots were prepared in the absence of bmercaptoethanol. **(D)** Thermal stability of HuPrP(23–230) wt and E200K probed by the relative change in the ellipticity at 220 nm as a function of temperature. Insert: Far-UV CD spectrum of HuPrP(23–230) wt and E200K. For panels B and C, samples were run in consecutive sequences in big gels which were cut after transfer for incubation with different antibodies and peptides incubation, as described in the manuscript.

## Supporting information

S1 File Uncropped blots for Figs 2, 3, 4 and 5. (PPTX)
