# Supplementary material for: Correction: Oxidation of Helix-3 Methionines Precedes the Formation of PK Resistant PrPSc
Source: PLoS Pathog. 2017 May 3;13(5):e1006293. doi: 10.1371/journal.ppat.1006293 (PMC5414941; doi:10.1371/journal.ppat.1006293)
Supplement: S1 File — (PPTX) [file ppat.1006293.s001.pptx]

## Slide 1
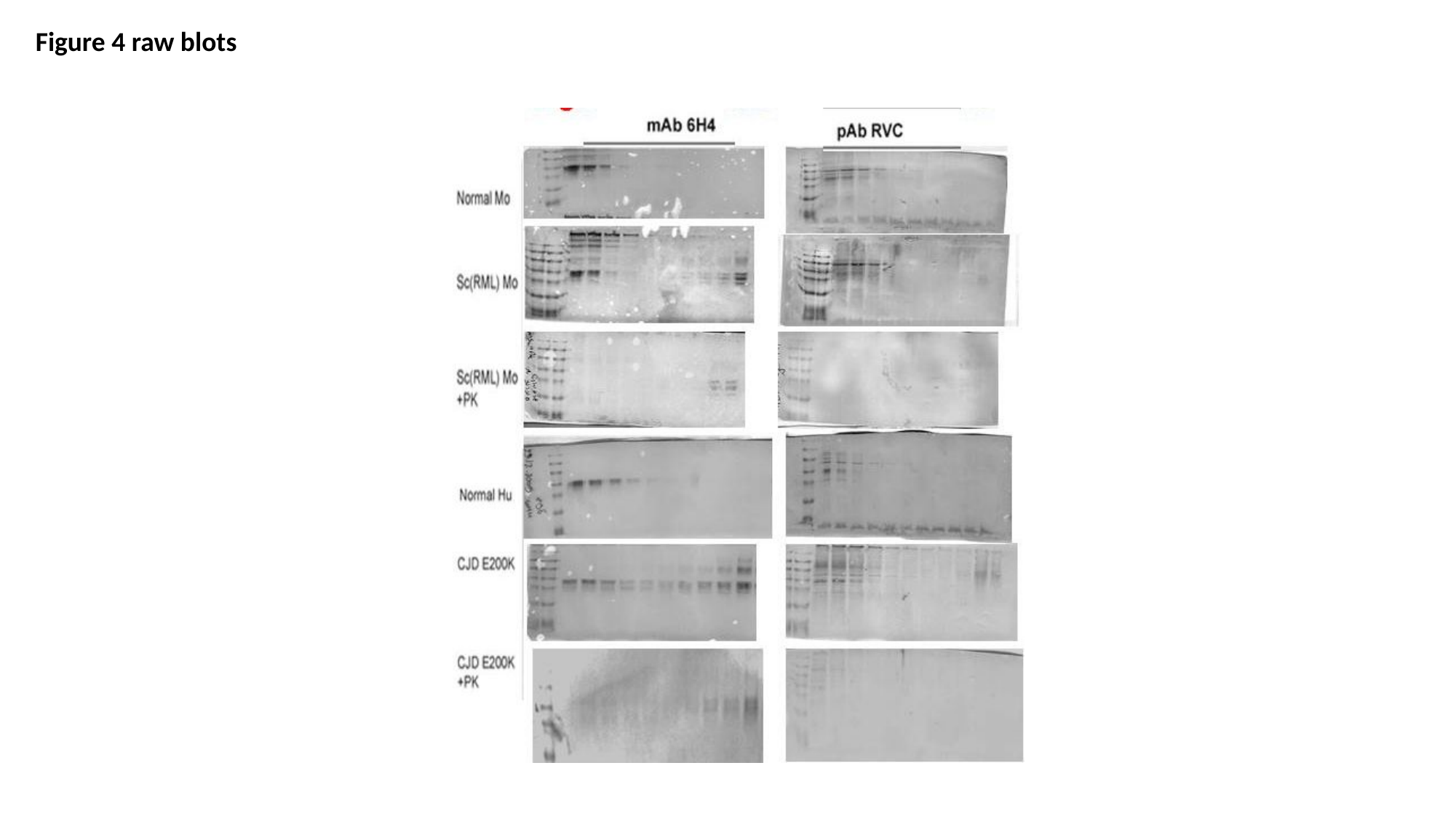

Figure 4 raw blots

## Slide 2
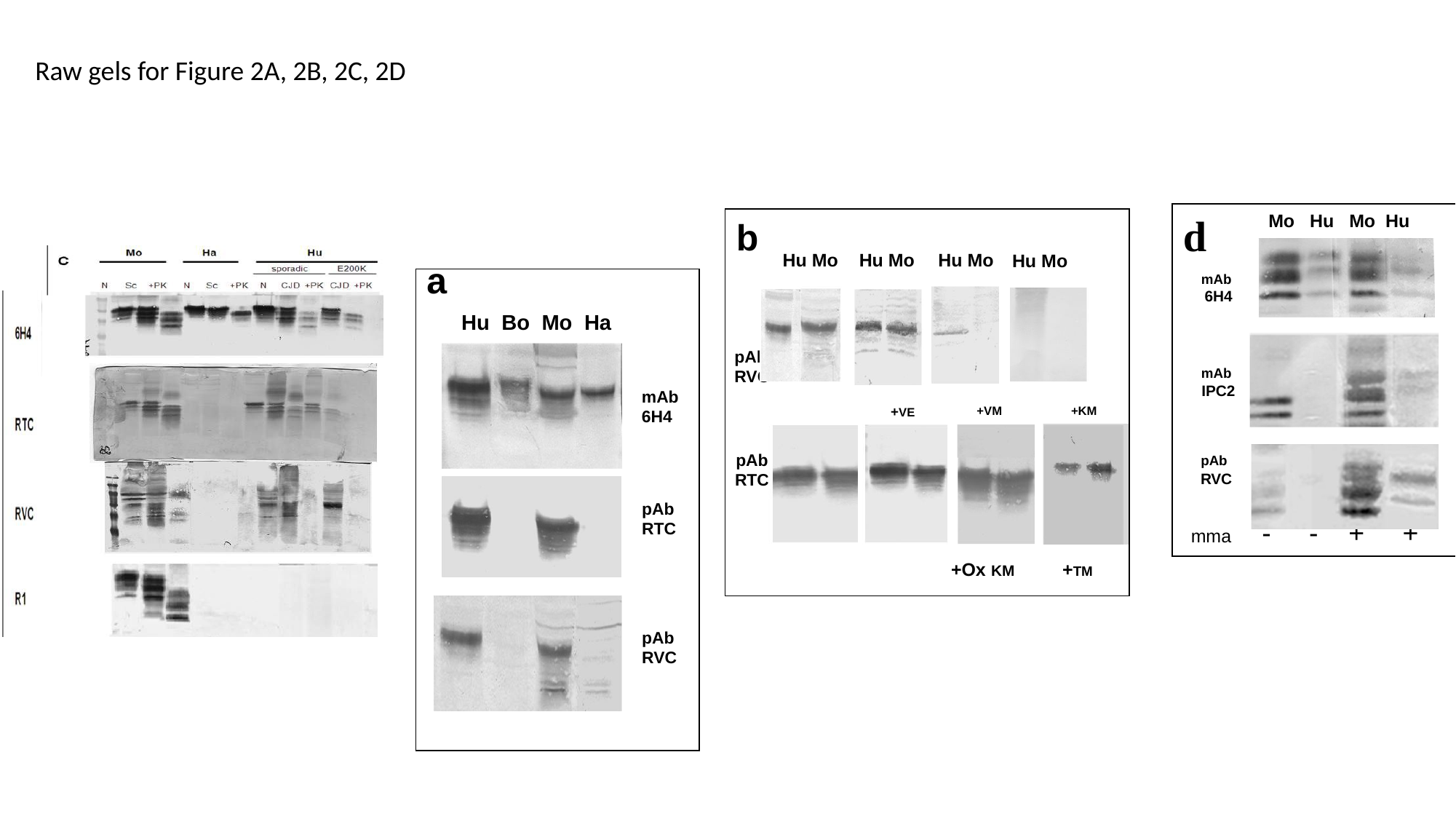

Raw gels for Figure 2A, 2B, 2C, 2D
Mo Hu Mo Hu
d
b
Hu Mo
Hu Mo
Hu Mo
Hu Mo
mAb
 6H4
a
Hu Bo Mo Ha
pAb
RVC
mAb
 IPC2
mAb
6H4
+VE
+VM
+KM
 pAb
RTC
pAb
RVC
pAb
RTC
mma - - + +
+Ox KM
+TM
pAb
RVC

## Slide 3
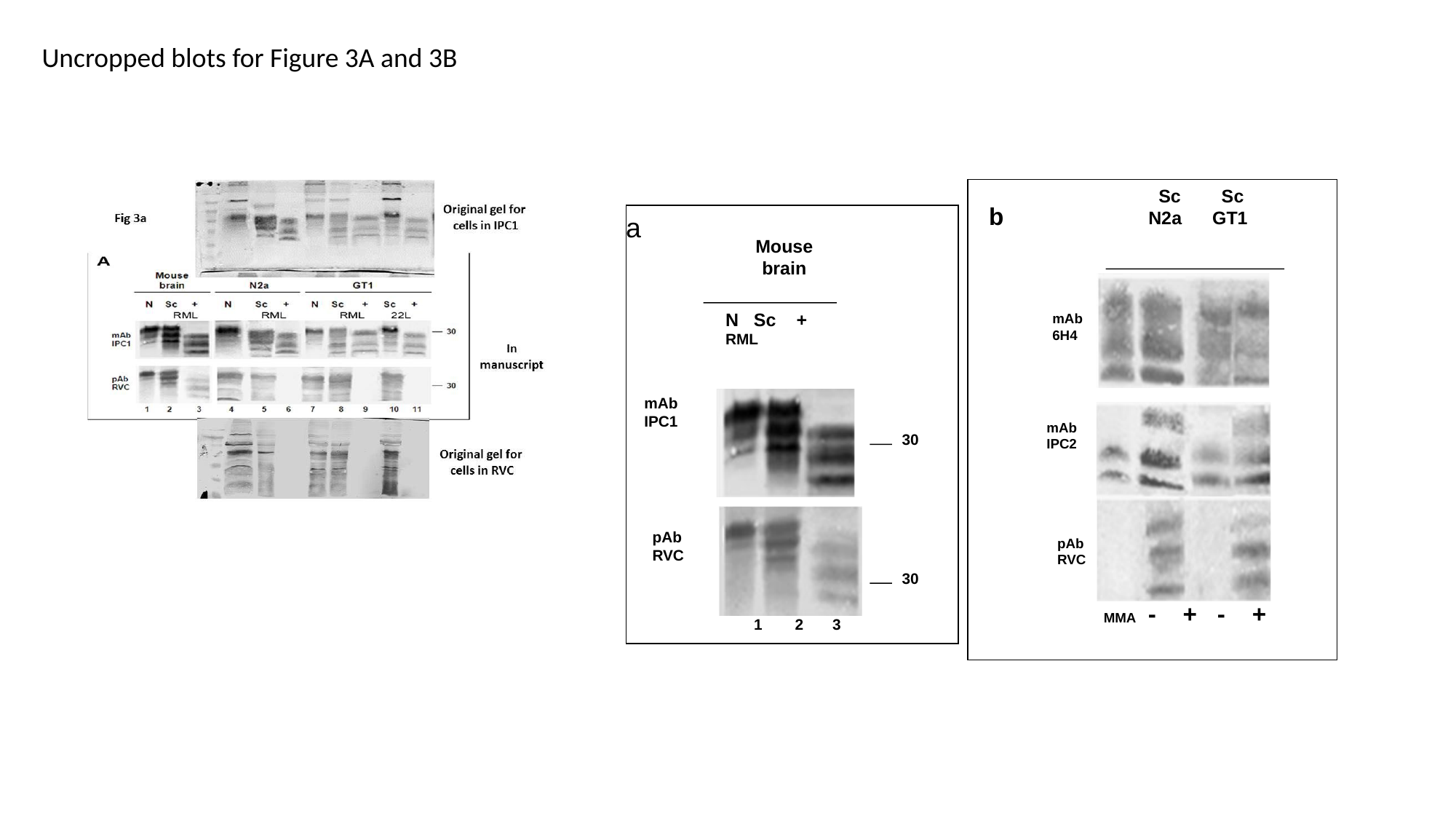

Uncropped blots for Figure 3A and 3B
 Sc Sc
N2a GT1
b
mAb
6H4
mAb
IPC2
pAb
RVC
MMA - + - +
a
Mouse
brain
N Sc +
RML
mAb
IPC1
30
pAb
RVC
30
2 3

## Slide 4
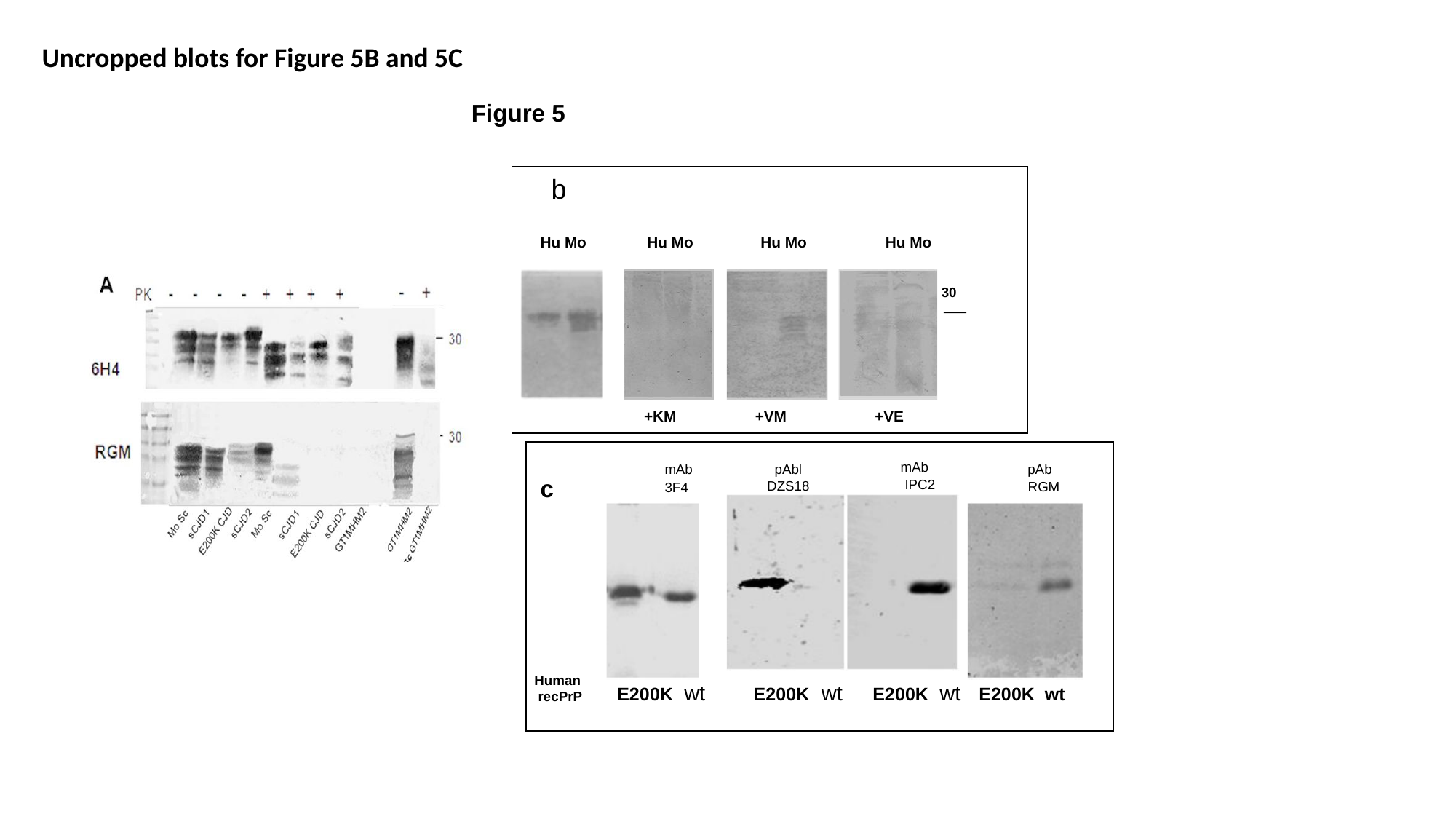

Uncropped blots for Figure 5B and 5C
Figure 5
b
Hu Mo
Hu Mo
Hu Mo
Hu Mo
30
+KM
+VM
+VE
mAb
 IPC2
pAb
 RGM
mAb
3F4
pAbl
DZS18
c
Human
 recPrP
E200K wt E200K wt E200K wt E200K wt
